# Increased efficiency of peripheral nerve regeneration using supercritical carbon dioxide-based decellularization in acellular nerve graft

**DOI:** 10.1038/s41598-024-72672-w

**Published:** 2024-10-10

**Authors:** Soon Jin Choi, Jeonghun Han, Young Ho Shin, Jae Kwang Kim

**Affiliations:** 1 Asan Institute for Life Sciences, Seoul, Korea; 2 DOF Inc., Hwaseong, Korea; 3 Department of Orthopedic Surgery, Asan Medical Center, University of Ulsan College of Medicine, 88, Olympic Road 43‐gil, Songpa‐gu, Seoul, 05505 South Korea

**Keywords:** Acellular nerve graft, Peripheral nerve regeneration, Supercritical carbon dioxide, Decellularized nerve, Neuroscience, Peripheral nervous system

## Abstract

Acellular nerve grafts (ANGs) are a promising therapeutic for patients with nerve defects caused by injuries. Conventional decellularization methods utilize a variety of detergents and enzymes. However, these methods have disadvantages, such as long processing times and the presence of detergents that remain on the graft. In this study, we aimed to reduce process time and minimize the risks associated with residual detergents by replacing them with supercritical carbon dioxide (scCO_2_) and compared the effectiveness to Hudson’s decellularization method, which uses several detergents. The dsDNA and the expression of MHC1 and 2 were significantly reduced in both decellularized groups, which confirmed the effective removal of cellular debris. The extracellular matrix proteins and various factors were found to be better preserved in the scCO_2_ ANGs compared to the detergent-ANGs. We conducted behavioral tests and histological analyses to assess the impact of scCO_2_ ANGs on peripheral nerve regeneration in animal models. Compared with Hudson’s method, the scCO_2_ method effectively improved the efficacy of peripheral nerve regeneration. Therefore, the decellularization method using scCO_2_ is not only beneficial for ANG synthesis, but it may also be helpful for therapeutics by enhancing the efficacy of peripheral nerve regeneration.

## Introduction

Peripheral nerve injuries that result in significant defects can cause pain and often leave patients with long-lasting neural deficits^1,2^. Clinical trials have been conducted to address nerve injuries and find solutions to the problem. Among all the approaches to repairing nerve injuries, autologous nerve grafting is considered the clinical “gold standard” and the most effective treatment^3^. However, this method has several restrictions, such as size mismatches, donor site morbidity, and lack of sufficient donor tissue^4–6^. Fresh cadaveric allografts can serve as an alternative to nerve autografts, but they require the host to undergo immunosuppression^7^. Therefore, researchers have developed and explored the use of synthetic conduits and decellularized nerve grafts as alternatives to bypass these limitations^8^. Decellularization of nerve grafts is a process that involves the removal of cells through chemical, physical, and enzymatic treatments. These methods can alter the properties of cellular components, thereby reducing the immunological response to ANGs^9,10^.

Decellularization is achieved through various techniques, with chemical detergents being widely used due to their potent ability to remove the cell nucleus during the decellularization process^11,12^. Particularly, detergents such as sulfobetaine (SB) and sodium dodecyl sulfate (SDS) are known to solubilize cell and nucleic membranes; however, they can also denature proteins and disrupt the ECM ultrastructure as well as the levels of various cytokines and growth factors^13–15^. Additionally, while some decellularization methods may be relatively short, extended washing processes are necessary to eliminate any remaining detergent^16,17^.

Due to concerns about the toxicity of chemicals, methods for physically removing the ECM are being developed. In particular, methods of physical treatments include freeze–thaw methods using liquid nitrogen and decellularization methods with scCO_2_^18^. Liquid nitrogen procedures alternate between freezing temperatures of approximately − 80 °C and biological temperatures of 37 °C. However, cell nuclei may remain even after such processes. Therefore, there is a possibility that the removal of genetic material is insufficient, potentially leading to an immune rejection reaction^19^. In contrast, the supercritical decellularization method does not require a washing process to remove chemicals because the solvent is released quickly due to the diffusivity of CO_2_ and does not remain in the tissue. Additionally, since CO_2_ is nonpolar, a process is performed to remove polar phospholipid cell membranes by adding ethanol. It has been reported that cell nuclei and membranes are cleanly removed, making them suitable for transplantation^18^.

In addition, scCO_2_ is known for being non-toxic, inexpensive, inert, and environmentally friendly. With a mild critical temperature of 31.1 °C, it can function effectively at normal body temperatures^16,20,21^. The decellularization process using scCO_2_ has the advantage of providing a much faster treatment time compared to water, taking only a few hours instead of several days^22^. Moreover, avoiding the use of detergents also reduces damage to the ECM and minimizes their toxic effects^23^. For this reason, scCO_2_ induced decellularized ECM has recently attracted attention as a potential therapeutic material in tissue engineering and regenerative medicine. Decellularization by scCO_2_ has been reported in several tissues; however, only a limited number of studies have applied scCO_2-_ANGs for peripheral nerve regeneration. Moreover, while a decellularization technique that utilizes a combination of detergent and supercritical extraction has been reported^24^, there have been few studies that applied scCO_2_ ANGs to animal models using solely the supercritical extraction method, without the use of detergent. Although it has been reported that angiogenesis-related factors are well preserved in decellularized heart ECM using the supercritical method^25^, no study has yet examined the preservation of growth factors and peripheral nerve regeneration in scCO2 ANGs transplanted in animal models.

**Fig. 1:**
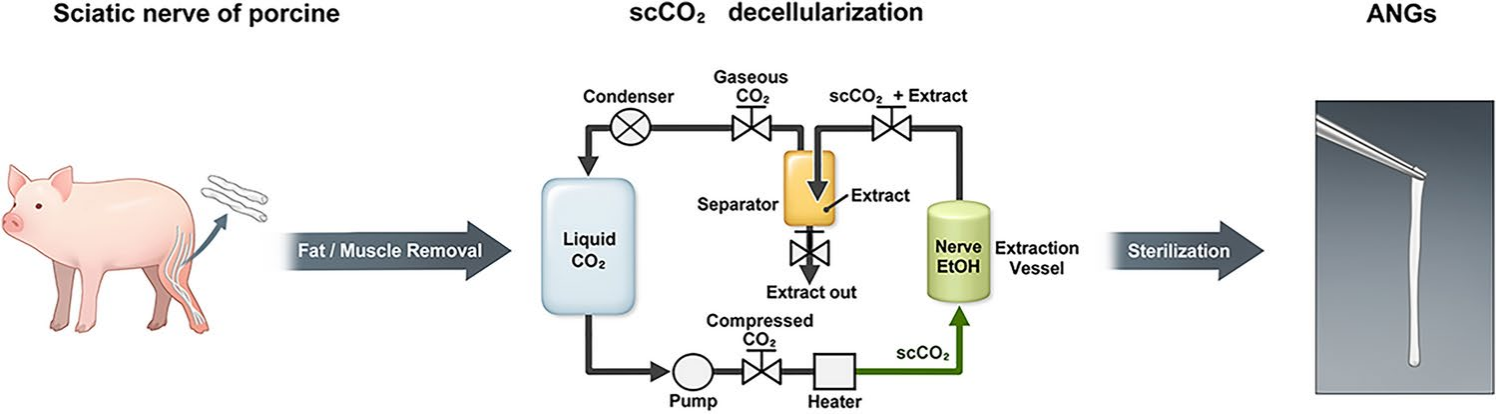
Preparation process of ANGs using supercritical CO_2_ (scCO_2_) decellularization technology.

Therefore, we conducted an experiment to decellularize porcine nerve tissue using supercritical fluid technology, which dissolves polar groups in tissues. Specifically, we sought to construct scCO_2_ ANGs without the use of any detergents (Fig. 1). Our ultimate objective was to completely remove the nuclei from cells while retaining the diverse array of growth factors, angiogenic factors, and ECM components present in the ANGs, in order to facilitate the regeneration of the peripheral nerve. To evaluate the degree of decellularization, we performed DNA quantification, DAPI, and H&E staining, as well as cytokine assays and quantification of ECM components in each group. In addition, we explored the potential of a novel scCO_2_ ANG as an alternative to the current ANGs that rely on detergents by assessing its efficacy in promoting peripheral nerve regeneration through in vivo studies.

## Results

### Evaluation of cell removal from decellularized nerves by scCO_2_

**Fig. 2:**
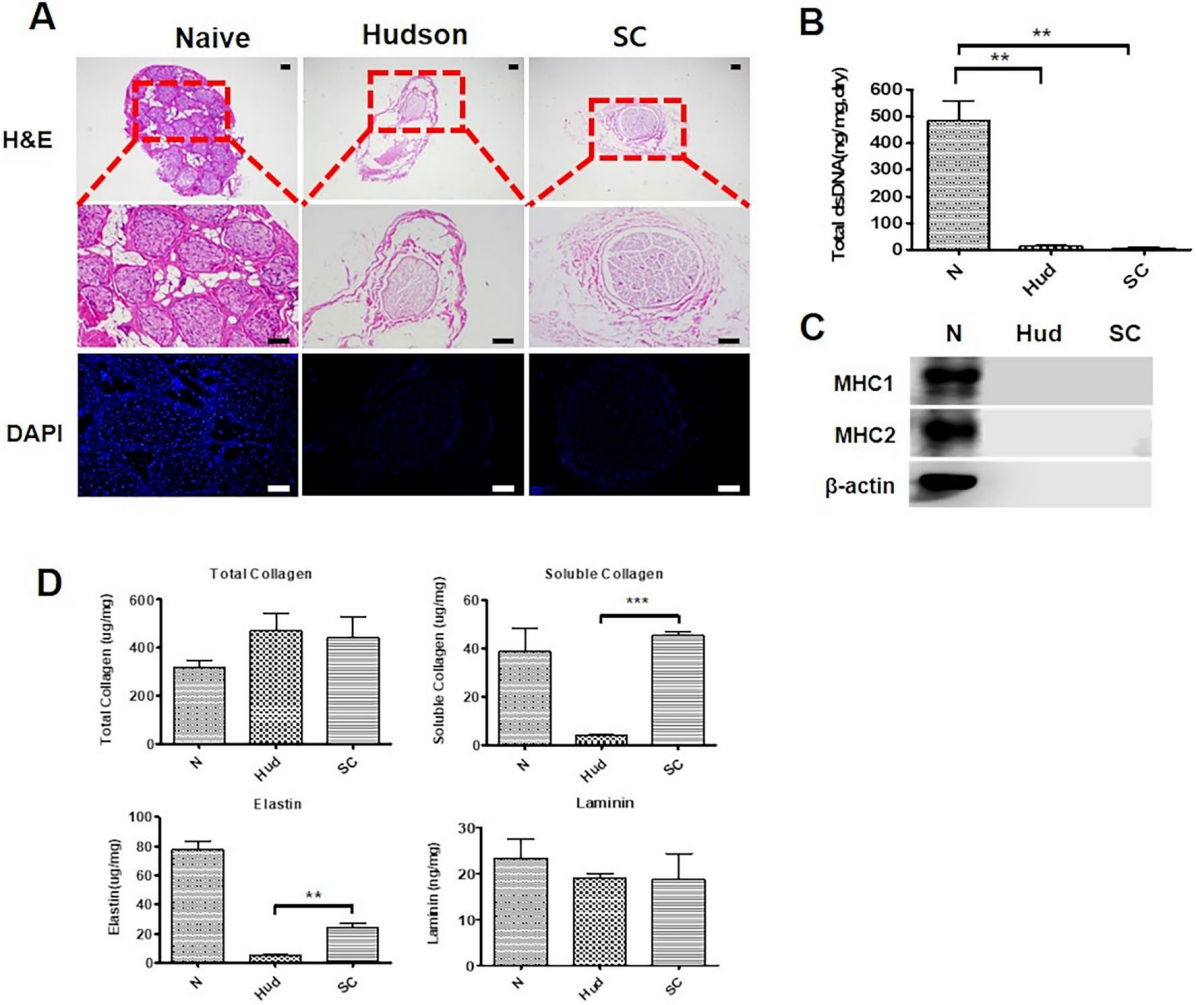
Characterization of scCO_2_ ANGs. **A** The Naive tissues and Decellularization of Hudson, and scCO_2_ ANGs were verified by detecting the absence of cell nuclei using HE (40´, 100´) and DAPI (100´) staining. Scale bar (black and white) = 100 μm. **B** The total amount of dsDNA was confirmed in the Native, Hudson and scCO_2_ ANGs. Each experiment was replicated three times or more. **C** MHC1, MHC2, and β-actin were analyzed by Western blotting. **D** The evaluation for the preservation of extracellular matrix (ECM) components, including total collagen, soluble collagen, elastin, and laminin contents, was quantified in N (native), Hudson, and scCO_2_ ANGs. Each experiment was replicated three times or more. The mean ± SEM of the Hudson group and the SC group were compared using a Student’s t-test (**p < 0.01, ***p < 0.001).

To investigate the benefits of scCO_2_ ANGs, we compared it with different decellularization methods, including scCO_2_ with ethanol and Hudson-ANGs (Fig. 2). To confirm the extent of cell removal, we verified the presence of cell nuclei through H&E and DAPI staining. In native nerve tissues, several fascicles were visible within the outermost epineurium of the nerve, and many types of cells, such as Schwann cells, adipocytes, and fibroblasts, were observed as well. On the other hand, the cell nucleus completely disappeared in both the scCO_2_ ANGs and Hudson ANGs (Fig. 2A). The levels of resident dsDNA were 14.6 ± 2.4 and 6.7 ± 2.9 ng/mg in the Hudson-ANGs and scCO2-ANGs, respectively (Fig. 2B). In a previous study, the decellularization criteria required less than 50 ng/mg dsDNA per mg of ECM dry weight and the absence of visible nuclei^10^. Both Hudson and scCO_2_ ANGs met the criteria for decellularization. The presence of cell debris in the ECM, which can trigger immune responses, was confirmed. MHC1 and MHC2, which are components of the cell membrane, as well as beta-actin, a cytoplasmic protein, were found to be present at high levels in the native tissues, but not in Hudson and SC nerves (Fig. 2C and Supplementary Fig. 1). The protein analysis results were consistent with the results of the quantitative analysis of dsDNA.

### Evaluation of ECM components

To assess the preservation of ECM in decellularized nerve tissue, we analyzed the levels of major proteins including collagen, elastin, and laminin. The total collagen content was determined by adding the soluble and insoluble fractions of collagen. The total collagen content was 320 ± 28.2 ug/mg in the Native group, 470 ± 72.0 ug/mg in the Hudson ANGs, and 442 ± 86.5 ug/mg in the scCO_2_ ANGs. The amount of soluble collagen was 38.8 ± 9.1 ug/mg in the Native group, 4.2 ± 0.03 ug/mg in the Hudson ANGs, and 45.5 ± 1.3 ug/mg in the scCO_2_ ANGs (Fig. 2D). There was no significant difference in total collagen between the groups; however, the level of soluble collagen in the scCO_2_ ANGs was significantly higher than that in the Hudson ANGs. The increased collagen content of the decellularized graft compared to the natural tissue was due to the removal of cells and fat during the decellularization process, as well as a decrease in the total weight of the decellularized tissue. Additionally, the amount of elastin was 77.5 ± 6.0 ug/mg in the Native group, 5.3 ± 0.1 ug/mg in the Hudson ANGs, and 24.3 ± 2.6 ug/mg in the scCO_2_ ANGs. The elastin content decreased in both the Hudson and scCO_2_ ANGs compared to the Native group, while the elastin content in the scCO_2_ ANGs was significantly higher than that in the Hudson group. The amount of laminin was 23.3 ± 4.2 ng/mg in the Native group, 19.0 ± 1.0 ng/mg in the Hudson ANGs, and 18.6 ± 5.6 ng/mg in the scCO_2_ ANGs. There was no significant difference in the laminin content among the groups (Fig. 2D).

### Angiogenic and growth factors preservation

**Fig. 3:**
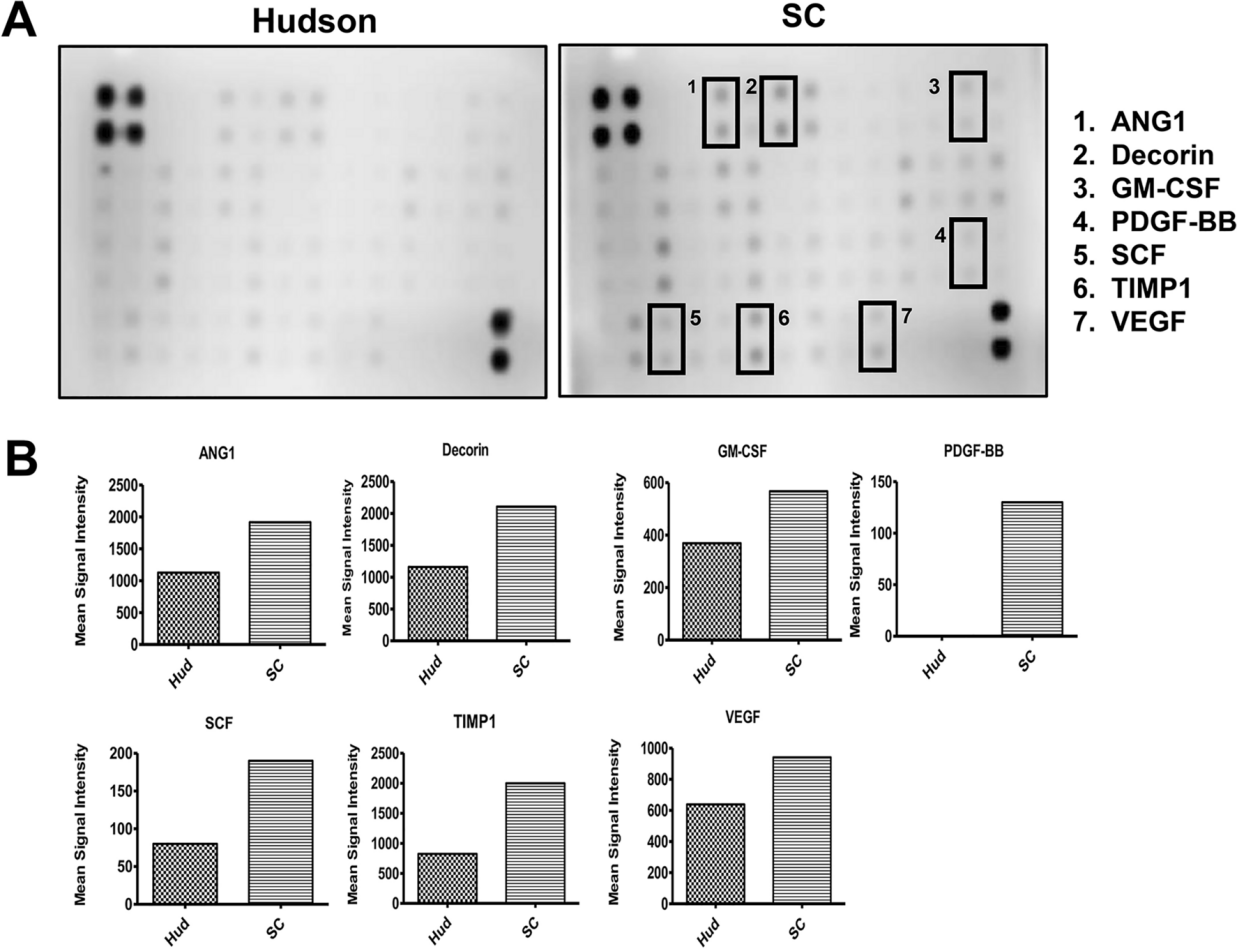
Evaluation of cytokine preservation. **A** 48 cytokine array image of Hudson and scCO_2_ ANGs. Black box means ANG1: 1, Decorin: 2, GM-CSF: 3, PFDG-BB: 4, SCF: 5, TIMP1: 6, VEGF: 7 spots. **B** Comparison of the mean signal intensities for each individual antigen-specific antibody spots among array images.

To confirm the preservation of various factors that can facilitate nerve regeneration after transplantation, we semi-quantitatively evaluated 48 cytokine targets. The factors related to nerve regeneration were higher in the SC group compared to the Hudson group. The levels of angiopoietin 1 (Ang1), Decorin, Granulocyte–Macrophage Colony-Stimulating Factor (GM-CSF), Platelet-Derived Growth Factor BB (PDGF-BB), Stem Cell Factor (SCF), Tissue Inhibitor of Metalloproteinase 1 (TIMP-1), and Vascular Endothelial Growth Factor (VEGF), which promote nerve regeneration after injury, were higher in the scCO_2_ ANGs than in the Hudson ANGs (Fig. 3).

### Cytotoxicity

**Fig. 4:**
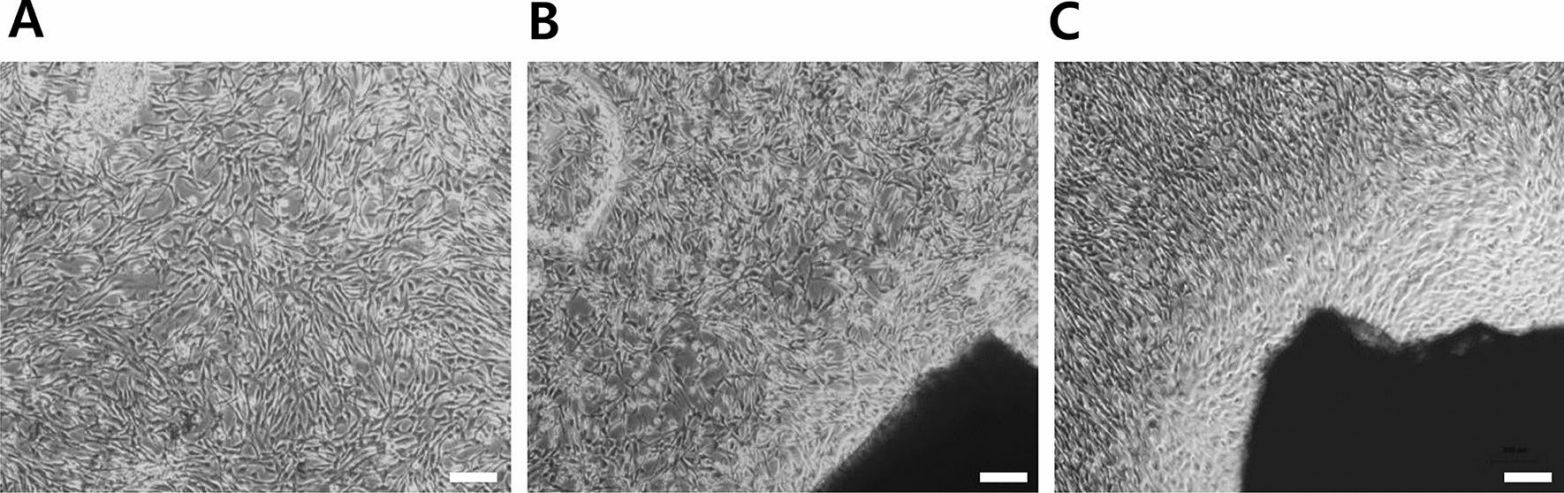
Contact cytotoxicity assays for the decellularized nerves after 48 h of culture with hSCs. The images illustrate that the decellularized nerve segments were non-cytotoxic to hSCs. **A** Collagen gel (control). **B** Decellularized nerve segment using the Hudson method. **C** Decellularized nerve segment using scCO_2_ method. scale bar, 200 μm.

The analysis of the contact cytotoxicity plates revealed that human Schwann cells (hSCs) had successfully adhered and made contact with all of the decellularized nerve segments tested in this study. In both the Hudson and scCO_2_ ANGs, no areas of morphological alterations or cell lysis were observed. Collagen alone served as the negative control, which exhibited no cytotoxicity (Fig. 4).

### Video gait angle analysis

**Fig. 5:**
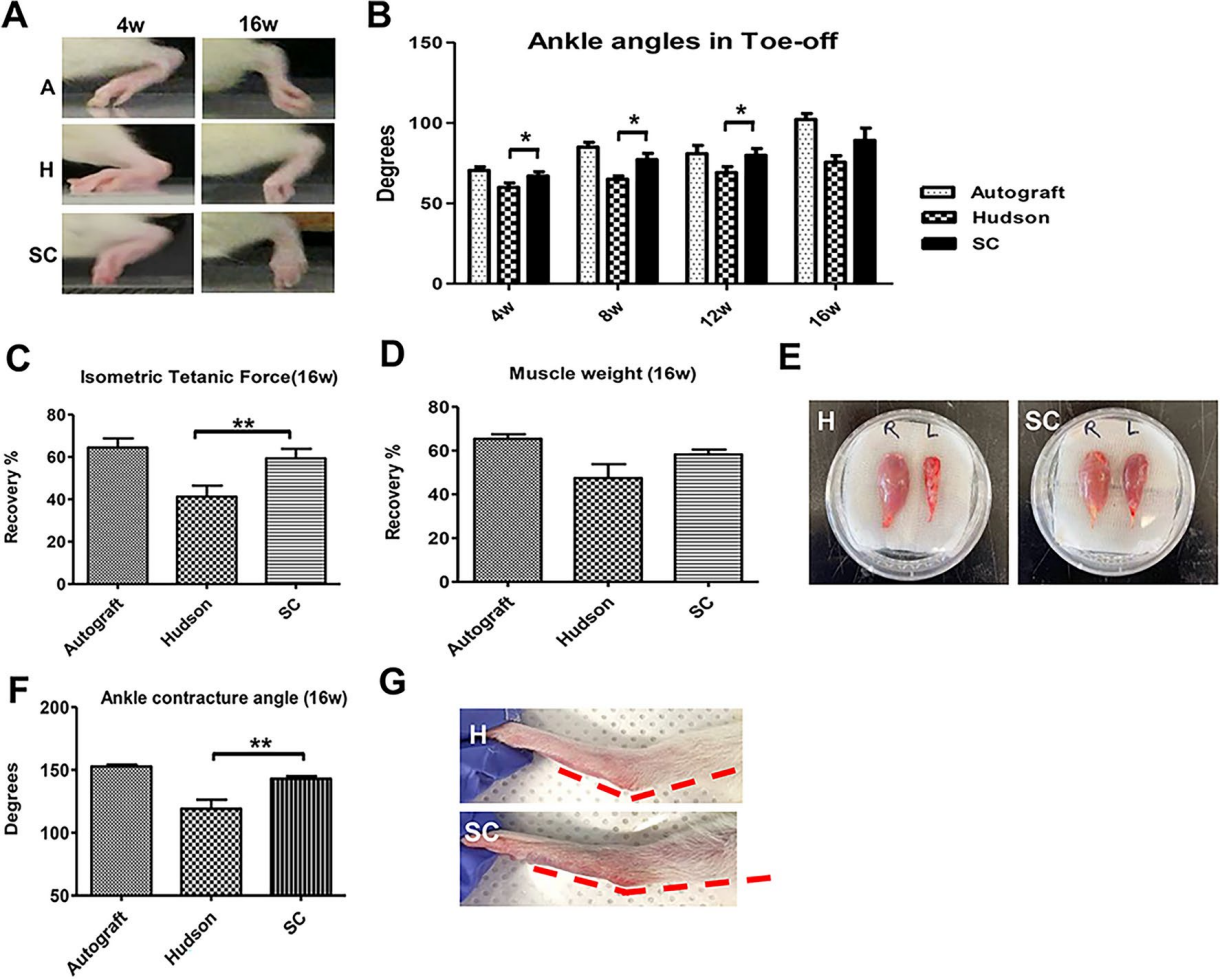
Evaluation of functional recovery in vivo*.***A**, **B** Analysis of ankle angles during the toe-off phase of gait in videos taken at 4, 8, 12, and 16 weeks after implantation. The angles were measured in the autograft, Hudson, and scCO_2_ ANGs up to 16 weeks. **C** Measurement of the isometric tetanic force and recovery rate at post-operative 16 weeks. **D**, **E** Comparison of muscle weight and recovery rate at post-operative 16 weeks. **F**, **G** Measurement of ankle contracture angle at postoperative 16 weeks. SD rats (N = 9) were used. The number of replicates is nine. The mean ± SEM of the Hudson and the scCO_2_ ANGs groups were compared using a Student’s t-test (*p < 0.05, **p < 0.01).

According to a previous study^26^, there is a strong correlation between toe-off phase angles and sciatic nerve regeneration. We therefore assessed the effect of ANGs on sciatic nerve regeneration by measuring the angle of the video gait. The mean ankle angles during the toe-off phase of the Autograft group were 70.3 ± 2.3, 84.7 ± 3.2, 80.7 ± 5.4, and 102 ± 3.9 degrees at 4, 8, 12, and 16 weeks, respectively. The Hudson ANGs showed significantly lower ankle angles compared to the autograft group at 4, 8, 12, and 16 weeks, measuring 59.7 ± 2.9, 64.8 ± 2.1, 69.0 ± 3.9, and 75.4 ± 4.3, respectively. The scCO_2_ ANGs showed higher ankle angles than the Hudson group at 4, 8, 12, and 16 weeks, measuring 66.8 ± 2.7 (*p* = 0.048 vs. Hudson), 77.0 ± 4.2 (*p* = 0.018 vs. Hudson), 79.78 ± 4.4 (*p* = 0.045 vs. Hudson), and 89.0 ± 8.18 (*p* = 0.081 vs. Hudson), respectively (Fig. 5A,B).

### Isometric tetanic force and muscle weight

The isometric tetanic force is a crucial tool for evaluating muscle strength after nerve recovery or injury^27^. At 16 weeks, the Autograft, Hudson, and scCO_2_ ANGs showed a percentage recovery of the isometric tetanic force in the TA of 65.3% ± 2.1%, 47.4% ± 6.3%, and 58.2% ± 2.2%, respectively. The scCO_2_ ANGs showed a significantly higher recovery rate compared to the Hudson ANGs at 16 weeks (*p* = 0.009; Fig. 5C). After analyzing the tetanic force, the TA muscles were harvested to measure their weight. The percentage of muscle weight recovery in the TA was 64.4% ± 4.3% for the Autograft group, 41.2% ± 5.1% for the Hudson ANGs, and 59.3% ± 4.5% for the scCO_2_ ANGs. The scCO_2_ ANGs showed a higher recovery rate than the Hudson group at 16 weeks, albeit without statistical significance (Fig. 5D,E).

### Contracture ankle angle

The greater ankle contracture angle is known to indicate better nerve regeneration; therefore, the analysis of the ankle contracture angle has been used as an assessment method for sciatic nerve regeneration^28^. At postoperative 16 weeks, the mean ankle contracture angles for the Autograft, Hudson, and scCO_2_ ANGs were 152.7 ± 1.5, 120.1 ± 6.5, and 142.9 ± 2.1, respectively. The Hudson ANGs showed significantly lower contracture angles compared to the autograft group (*p* = 0.0003), while the scCO_2_ ANGs showed significantly higher angles than the Hudson group (*p* = 0.005; Fig. 5F,G).

### Analysis of toluidine blue and immunohistochemistry staining

**Fig. 6:**
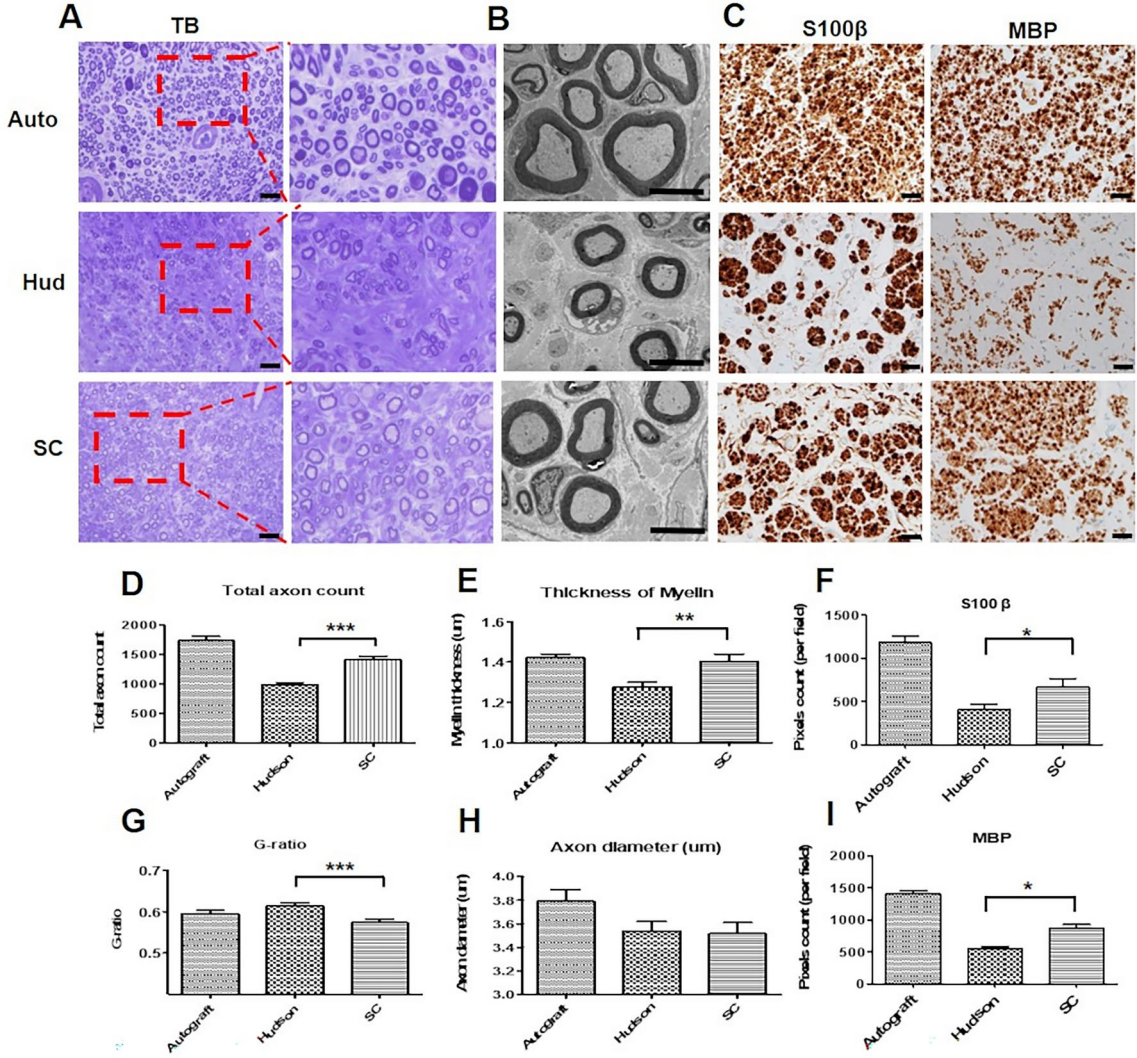
Histological evaluation of axon regeneration and SC myelination. **A** Toluidine blue staining of the autograft, Hudson, and scCO_2_ ANGs at postoperative 16 weeks. Scale bar: 50 μm. **B** Electron microscopy images of the autograft, Hudson, and scCO_2_ ANGs. Scale bar: 5 μm. **C**, **F**, **I** Immunostaining and analysis of myelination markers S100β and MBP. Scale bar: 50 μm. **D**, **E** Total axon count and Thickness of myelin are shown. **G**, **H** G-ratio and Axon diameter are shown. SD rats (N = 9) were used. The number of replicates is nine. The mean ± SEM of the Hudson ANGs and the scCO_2_ ANGs groups were compared using a Student’s t-test (*p < 0.05, **p < 0.01, ***p < 0.001).

At postoperative 16 weeks, toluidine blue staining was used to directly evaluate the regeneration of sciatic nerve axons in each group. The histological structures of axons and nerve fibers are shown in Fig. 6A. The total number of myelinated axons in the Autograft, Hudson, and scCO_2_ ANGs were 1739.6 ± 67.6, 986.2 ± 38.1, and 1412.0 ± 56.6, respectively. The scCO_2_ ANGs showed a significantly higher total axon count than the Hudson ANGs (*p* < 0.001; Fig. 6D). The specific markers of SCs S100 calcium-binding protein (S100β), and MBP were analyzed through IHC staining (Fig. 6C). The pixel counts for the expression of S100β in the Autograft, Hudson, and scCO_2_ ANGs were1181.0 ± 75.6, 406.4 ± 58.8, and 665.3 ± 98.2, respectively. The pixel counts for the expression of MBP were 1405.0 ± 47.7, 553.6 ± 34.2, and 870.9 ± 54.5 in the Autograft, Hudson, and scCO_2_ ANGs, respectively. The scCO_2_ ANGs showed significantly higher expression levels of both S100β and MBP than the Hudson-ANGs (both *p* < 0.05; Fig. 6F,I). The markers of blood vessels CD31 and CD34, were analyzed using IHC staining. The scCO2 ANGs showed higher expression levels of both CD31 and 34 than the Hudson ANGs, but not significantly (data not shown).

### Analysis of transmission *electron* microscopy (TEM)

At postoperative 16 weeks, we analyzed the structures of myelinated axons using TEM (Fig. 6B). To further characterize myelin regeneration, we assessed the thickness of myelin, g-ratio, and axon diameter in each group. The thickness of myelin in the Autograft, Hudson, and scCO2-ANGs was 1.42 ± 0.01, 1.27 ± 0.02, and 1.40 ± 0.03, respectively. The scCO2 ANGs exhibited significantly thicker myelin than the Hudson ANGs (*p* = 0.0037; Fig. 6E). The g-ratios, representing the ratio of the inner axon diameter to the outer axon diameter of the myelin sheath, were 0.595 ± 0.007, 0.613 ± 0.007, and 0.573 ± 0.008 in the Autograft, Hudson, and scCO_2_ ANGs. The scCO_2_ ANGs showed a significantly lower g-ratio than the Hudson group (*p* = 0.0003; Fig. 6G). Moreover, the axon diameters in the myelin of the Autograft, Hudson, and scCO_2_ ANGs were 3.792 ± 0.098, 3.538 ± 0.080, and 3.517 ± 0.093, respectively (Fig. 6H). However, there was no significant difference between the scCO_2_ ANGs and the Hudson ANGs.

## Discussion

During the process of decellularizing nerves, it is crucial to find the optimal method that balances the complete removal of cellular components with the preservation of the ECM structure in order to improve the efficiency of peripheral nerve regeneration^10,29^. Hudson et al. developed the novel detergents formula for the engineering of ANGs after a comprehensive examination to date of the effects of detergents on peripheral nerve tissue and they reported their method showed superior peripheral nerve regeneration compared to the previous decellularized method^30,31^. However, detergent-based methods, including the use of ionic detergents like the Hudson method, can cause greater disruption of the ECM and more toxic side effects if not completely removed during the process. Therefore, recent studies have explored ways to reduce the detergents by adding DNAase and RNAase^29^. In addition, Kim et al. demonstrated that the decellularization by the osmotic effects of hypotonic and hypertonic solutions and detergents was superior to the Hudson method^32^. Our study also showed that scCO_2_ ANGs without detergent were superior to Hud ANGs.

Acellular nerve allografts were initially developed as an alternative to autologous nerve transplants. Some studies have reported that there is no difference in outcomes between ANGs and reverse autografts^33,34^. However, these studies were limited to short gaps, and for longer gap distances, the results of autograft transplantation were superior to ANGs^35^. Mackinnon et al. investigated axonal regeneration across the lengths of ANGs in rat animal models and the results revealed that as graft length increased, both axonal regeneration and functional recovery decreased, with isograft performing better than ANGs^36^. Our results indicated that the functional and histological outcomes of reverse autograft were still superior to ANGs decellularized from porcine nerve tissue in rat animal models.

In this study, we sought to develop a scCO_2_ decellularization method that reduces DNA content while preserving ECM components and enhancing the regeneration of peripheral nerves in animal models. Previous studies have reported that ECM decellularized by scCO_2_ yields superior outcomes in terms of skin regeneration and angiogenesis compared to chemical-based decellularization methods^25,37^. However, studies on decellularization using scCO_2_ are still insufficient; particularly, few studies have evaluated the degree of peripheral nerve regeneration by scCO_2_ ANGs without chemical detergents in rat models.

Therefore, we established scCO_2_ ANGs and evaluated their effectiveness in promoting peripheral nerve regeneration, comparing them with traditional Hudson ANGs.

We initially examined the characterization of scCO_2_ ANGs in terms of their ability to remove cells while preserving ECM structures and molecules in comparison to Hudson ANGs. The presence of cellular components, such as DNA and RNA, within the ECM can trigger an immune response in the body^38^. Previous research has indicated that to prevent negative reactions caused by residual nucleic acids, ECM should contain less than 50 ng dsDNA per dry weight and should not have any visible nuclei^10^. In our study, we demonstrated the complete removal of nuclei and immunogenic contents, including MHC1 and MHC2 in which a representative protein involved in immune rejection, in from scCO_2_ ANGs. In addition, collagen and elastin play crucial roles in the ECM, constituting approximately 49% of the total protein in the peripheral nerve, and are known to contribute to the mechanical stability of the ECM^39–41^. Water-soluble collagen is known to be more susceptible to acid-pepsin degradation compared to insoluble collagen. Furthermore, not only is collagen affected by EDTA during the decellularization process, but it is also lost during the subsequent washing stages^42,43^. We quantified the amount of total and soluble collagen and elastin in the ECM and confirmed that the scCO₂ ANGs preserved the soluble collagen and elastin content better than those prepared using the Hudson method. Additionally, contact cytotoxicity assay showed that SCs that were directly in contact with ANGs did not undergo cytolysis and maintained their phenotypic structure well, indicating that decellularized ANGs are not cytotoxic.

To verify the preservation of other factors related to peripheral nerve regeneration, we investigated whether scCO₂ ANGs maintained various factors compared to Hud-ANGs. We observed that scCO₂ ANGs preserved various factors, including ANG1, Decorin, GM-CSF, PDGF-BB, SCF, TIMP1, and VEGF, to a greater extent. Ang1 and Decorin have been reported to act as a regulator of angiogenesis an angiogenic factor promoting their stabilization and maturation^44,45^. Decorin has been reported to act as a regulator of angiogenesis^46^. GM-CSF is a glycoprotein that promotes wound healing and exerts neuroprotective effects^47,48^. PDGF-BB plays a central role in damaged nerves by stimulating the migration of endothelial progenitor cells and MSCs^49,50^. SCF is also known to induce the differentiation and proliferation of neural stem cells^51,52^. TIMP-1 promotes VEGF-induced neovascularization in the retina and is known as one of the most up-regulated genes in injured nerves^53^. VEGF plays a crucial role in regulating vasculogenesis and has been shown to impact nerve regeneration^54,55^. In general, growth factors are essential for repairing and regenerating tissues and are involved in wound healing and tissue remodeling^56,57^. In particular, angiogenesis-related factors are known to contribute to vascular neogenesis and are related to axon growth for nerve regeneration and functional recovery by contributing to the migration and invasion of SCs^58,59^. Supercritical decellularized nerve tissue has been found to have more mechanisms associated with the VEGF pathway, which induces efficient signaling for angiogenesis, compared to chemically decellularized nerve tissue. This would lead to a shorter time for angiogenesis and enhance the efficiency of peripheral nerve regeneration.

Our in vivo studies using video gait angle analysis, isometric force assays, muscle weight measurement, and contracture angle analysis revealed that animals in the scCO₂ ANG group had superior results in terms of peripheral nerve regeneration compared to the control group that used Hudson-ANGs. Consistent with the results of functional recovery, toluidine blue staining, analysis of myelin by TEM, and the expression patterns of S100β and MBP in regenerated peripheral nerves showed that scCO₂ ANGs effectively promoted myelination.

Some similar studies have also been published recently. Wei et al. investigated the potential of supercritical extraction technology in a sciatic nerve defects model and further increased its efficiency by adding detergent to the decellularization process^24^. Le et al. demonstrated that nerves decellularized using scCO_2_, which is a new type of acellular nerve graft, were effective in peripheral nerve regeneration^60^. However, as Le et al. used a relatively mild injury model, additional investigation in models with more severe injuries is warranted. Furthermore, they did not examine the preservation of various growth factors related to peripheral nerve regeneration in nerves decellularized using scCO_2._

This study is limited in that we did not investigate the regeneration and functional recovery rate over time, which would have been useful for comparing the vascularization between scCO₂ ANGs and Hudson ANGs in the early post-implantation period. However, despite these limitations, this is the first study to analyze the residual various growth factors of scCO₂ ANGs without using any chemicals. The results showed that scCO₂ ANGs have significantly superior peripheral nerve regeneration compared to Hudson ANGs.

We hypothesize that the preservation of various factors in scCO₂ ANGs may have created a more conducive environment for nerve regeneration, resulting in enhanced regeneration. These findings indicate that the use of scCO₂ ANGs as implants holds promise for clinical applications as grafts to restore motor and sensory functions following nerve injuries. Moreover, scCO₂ ANGs are anticipated to emerge as a new alternative to autografts in peripheral nerve regeneration, in both preclinical and clinical settings.

## Conclusions

Our study shows that the scCO_2_ extraction method can be used to decellularize porcine peripheral nerves without the need for any detergents, which can be expected to retain higher levels of various factors that are useful in improving the efficiency of peripheral nerve regeneration after implantation.

## Material and methods

### Preparation of scCO_2_ ANG

Porcine peripheral nerve tissues were obtained from LEEGANAE, a livestock company (Gimje, Jeonbuk, South Korea), and nerve tissues with a diameter of approximately 2 mm were collected from the nerve tissues of multiple pigs. The scCO_2_ ANG was prepared in the order shown in Fig. 1. After removing the fat and muscle from the tissue, the nerves were washed with sterilized using 1X phosphate-buffered saline (PBS, Cat# L0615-500, BioWest, USA) for 30 min. scCO_2_ extraction was performed using equipment (DOF Inc.) consisting of a 0.3 L cylindrical vessel. The nerve tissue was immersed in 1 × PBS containing 1% antibiotics and placed on a 30 rpm rocker overnight. The CO_2_ flow was controlled using a gas flowmeter. EtOH (30–70%, Cat# 1.00983.5000, Sigma Aldrich, USA) was added to the vessel as a co-solvent. The pressure of the supercritical process was applied to nerve tissue at 200–300 bars. The processing time for scCO_2_ extraction is approximately 3 h. After the scCO_2_ extraction was complete, the nerve was washed twice with 1 × PBS for 15 min on a 30 rpm rocker. Hudson’s decellularization process was performed according to the previously published protocol^31^, and three different detergents were used to replicate the same incubation times and concentrations. Zwitterionic sulfobetaine (SB) 10 (Cat# S-1020) and 16 (Cat# S-1026) were purchased from AG Scientific (CA, USA). Triton X-200 was replaced with DOWFAX 2A1 (DOW Chemical, USA). All samples were sterilized using gamma irradiation at a dose of 25 kGy. The decellularized nerve tissue was stored at -80 °C for subsequent analysis and animal experiments.

### Histological analysis

After decellularization, the tissues were immersed in a 10% formaldehyde solution for 24 h and then embedded in paraffin using a tissue processor (Cat# 5903, SAKURA Finetek, Japan). The tissue samples were cut into 5 µm-thick slides. These slides were used for histological examinations in the future. For hematoxylin and eosin (H&E) staining, the tissue samples underwent deparaffinization in xylene for 3 min twice before being rehydrated in a reducing concentration gradient of ethanol (100%, 90%, 80%, 70%, and 0% v/v in water). The tissue samples were incubated in Mayer’s hematoxylin solution (Cat# S3309, Dako, USA) for 3 min, followed by two 3-min washes in distilled water to remove the excess stain before being incubated in the bluing reagent for 10–15 s. The tissue samples were then rinsed twice in distilled water for 3 min each time, followed by incubation in Eosin Y solution (Cat# 3610MIRA01, BBC Biochemical, USA) for 1 min, and subsequently washed with 95% ethanol. Finally, the tissue samples were dehydrated in three changes of 100% ethanol every 3 min and then mounted with mounting media (Cat# SL80-4, StatLab, USA). For staining with 4′,6-diamidino-2-phenylindole (DAPI), the tissue sections were deparaffinized and dehydrated as described above and then blocked with 4% BSA in 1X PBS buffer at room temperature for 60 min. Afterward, the tissue samples were rinsed twice with 1X PBS buffer for 3 min each time and subsequently mounted using a DAPI solution. HE and DAPI staining images were acquired using an inverted optical microscope for slide glass (CX33, OLYMPUS, Japan) and a fluorescence microscope (DM2500, LEICA, Germany), respectively.

### Determination of DNA content

DNA extraction was performed using the salting-out method. Briefly, about 5 mg of lyophilized nerve tissue was placed in a sterile microtube and minced. The tissue was pre-treated with proteinase K (Cat# 19131, QIAGEN, Germany) for 16 h at 56 °C. Then, 0.5 volumes of saturated NaCl (Cat# S9888, Sigma Aldrich, USA) were added to the lysed tissues. The sample was vigorously shaken and then centrifuged at 13,000 rpm for 10 min at 4 °C. The supernatant was transferred to a new tube and an equal volume of isopropanol (Cat# 19516, Sigma Aldrich, USA) was added and mixed. The mixed samples were incubated at RT for 30 min. Centrifugation was performed at 13,000 rpm for 10 min at RT to isolate DNA. After discarding the supernatant from the tube, the DNA pellet was air-dried for approximately 5 min. Next, the dried DNA was dissolved in UltraPure distilled water (Cat# 10977-015, Invitrogen, USA). To quantify dsDNA using a fluorescent reagent that binds directly to it, we analyzed the extracted DNA with the Qubit dsDNA HS assay kit (Cat# Q32854, Thermo Fisher Scientific, USA) following the manufacturer’s protocol. The Qubit Flex fluorometer (Cat# Q33327, Thermo Fisher Scientific, USA) was used to detect fluorescence signals and calculate the concentration of dsDNA. The total amount of dsDNA was normalized to the dry weight of the tissue. dsDNA was quantified from three different parts of nerve tissue for each group.

### Western blot

To confirm the clearance of cell debris in the decellularized nerve tissue, proteins were extracted from the tissue and analyzed through electrophoresis using specific antibodies for proteins found in cell membranes and cytoplasm. Each tissue sample was lysed with RIPA lysis buffer on ice by vortexing every 15 min for 1 h, and the supernatant was collected by centrifugation at 13,000 rpm for 15 min. Protein was quantified using the Bradford protein assay with Protein Assay Dye Reagent Concentrate (Cat# 5000006, Bio-Rad, USA). Then, 23 mg of denatured protein was separated using Laemmli buffer on a 10% Sodium Dodecyl Sulfate–Polyacrylamide Gel Electrophoresis (SDS-PAGE). The separated proteins were transferred onto an Immobilon-P transfer membrane (Cat# IPVH00010, Millipore, USA). After blocking with 1 × Tris-buffered saline with Tween 20 containing 5% skim milk (Cat# 1.15363.0500, Merck, USA) at RT for 1 h, the membranes were incubated with primary antibodies against MHC1 (1:1,000, Cat# SC-55582, Santa Cruz Biotechnology, USA), MHC2 (1:1,000, Cat# SC-59318, Santa Cruz Biotechnology, USA) and β-actin (1:1000, Cat# SC-47778, Santa Cruz Biotechnology, USA) overnight at 4 °C on a rocker. The membranes were washed on a rocker at RT for 1 h, with 1 × TBST being replaced every 15 min. The membranes were incubated with goat anti-mouse IgG secondary antibody (1:2,000, Cat# ADI-SAB-100-J, ENZO Life Sciences, USA) at RT for 1 h on a rocker. The membranes were washed on a rocker at RT for 1 h, with 1 × TBST being replaced every 15 min. Band signals were visualized using the C-DiGit Blot Scanner (Cat# 3600, LI-COR Biosciences, USA).

### ECM quantification

A hydroxyproline assay was conducted to measure the amount of collagen by using Sircol soluble and insoluble collagen assay kits (Cat# S1000, S2000, Biocolor, UK). Briefly, about 5 mg of each lyophilized tissue sample was incubated with acid-pepsin at 4 °C overnight to extract soluble collagen. To obtain insoluble collagen, the samples were immersed in a fragmentation reagent and placed in a homogenizer at 65 °C for 3 h; the supernatant was then collected by centrifugation at 12,000 rpm at 4 °C. Then, 100 µl of the collected supernatant was mixed with Sircol dye reagent and incubated at RT for 30 min, and then centrifuged at 12,000 rpm for 10 min. The pellets were washed with ice-cold acid salt wash reagent to remove any unbound dye and then centrifuged at 12,000 rpm for 10 min. Finally, the pellets were re-suspended in an alkali reagent, and the collagen content was measured by determining the optical density at 550 nm. The total amount of collagen was calculated by adding the quantities of both soluble and insoluble collagens. The elastin assay was performed as previously described^37^. The Fastin elastin assay kit (Cat# F2000, Biocolor, UK) was utilized to quantify the amount of elastin present in the tissue samples. To dissolve 5 mg of each lyophilized tissue sample, 750 L of 0.25 M oxalic acid was used at a temperature of 100 °C. Equal parts of the elastin precipitating solution were then added, and the mixture was vortexed for 15 min. The supernatant was drained and 1 ml of dye reagent was added after centrifugation at 10,000 g and 4 °C for 10 min. The samples were shaken for 90 min and then centrifuged at 10,000 g for 10 min. Each sample was treated with 250 L of dye dissociation reagent after removing the unbound dye. The absorbance was measured at a wavelength of 513 nm. In addition, an enzyme-linked immunosorbent assay (ELISA) was conducted to detect laminin in both native and decellularized nerves. The Porcine Laminin ELISA kit (Cat# MBS735627, MyBioSource, USA) was used to analyze the protein lysates. About 20 mg of each experimental group was used in this analysis. Laminin content was normalized to the wet weight of the tissue.

### Cytokine array

To verify the preservation of cytokines, the nerve tissues were lysed on ice for 1 h, and 45 μg of the extracted protein was quantified using the Bradford protein assay. The intensity of 48 porcine cytokines, including growth factors, in both native and decellularized samples, was measured using the Ray Bio C-Series Porcine Cytokine Array 1 kit (Cat#AAP-CYT-1, Ray Biotech, USA), which is a membrane-based antibody array. Target proteins in the sample bind to capture antibodies that are displayed in duplicate on membranes. The collected proteins were identified using biotinylated detection antibodies and chemiluminescent detection tools. The amount of analyte that is bound determines the signal production. Spot signal intensities were visualized and semi-quantified using the C-DiGit Blot Scanner. Positive control spots were used for normalization and to orient the arrays.

### Cytotoxicity analysis

Cytotoxicity analysis was performed according to the International Organization for Standardization (ISO) 10993-5, using the direct contact method as applied to hSCs. hSCs were obtained from ATCC (Manassas, VA) and cultured in DMEM with 10% FBS in a humidified atmosphere with 5% CO2. Confluent cells were subcultured after being detached with 0.25% trypsin and 0.05% ethylenediaminetetraacetic acid for 3 min. Each 5 mm fragment of ANGs was attached to the center of six-well tissue plates using rat tail collagen type I (#C3867, Sigma). hSCs were then seeded into at a density of 1.5 × 10^5^ cells/well and cultured at 37 °C with 5% CO2 for 48 h. The cells were rinsed with PBS and then fixed with 4% (v/v) paraformaldehyde-PBS (Biosesang, Gyeonggi, Korea) for 30 min. The Giemsa solution was diluted 1:20 in PBS before use and applied to each well for 1 h to stain the samples. After washing with PBS, each well was left to air dry. The plates were examined using an inverted microscope (CKX41, Olympus, Japan). Collagen type I was used as a negative control. Any changes in cell morphology were recorded.

### Surgical procedure

This study complied with the Animal Research: Reporting of In Vivo Experiments (ARRIVE) guidelines. All animal care and experimental procedures were approved by the Institutional Animal Care and Use Committee of Asan Medical Center and Ulsan University College of Medicine (approved No. 2021-13-341), and all the following methods were performed in accordance with the relevant guidelines and regulations.

Sprague–Dawley rats weighing 250–350 g (Orient Bio Inc., Seongnam, Korea) were randomly assigned to three groups: 9 in the autograft group, 9 in the Hudson group, and 9 in the supercritical carbon (SC) group. The porcine scCO_2_ ANGs were decellularized using the method described previously. The surgical sites on the left were shaved and sterilized using 75% ethanol after administering anesthesia. The skin was incised along the femoral axis, and the femoral muscle was dissected to expose the sciatic nerve. The left sciatic nerve was cut and constricted near the obturator muscle in the center of the thigh. Six mm of the nerve were removed after the nerve stump was cut. In the autograft group, the excised nerve segment was sutured after inverting the proximal and distal ends. For the ANGs group, the prepared decellularized nerves were sutured. All sutures were performed using 9–0 nylon (Ethicon, Somerville, NY) under a microscope. The skin incisions were then closed with wound clips.

### Video gait angle analysis

The recovery of the sciatic nerve is known to be correlated with the angle measured during the toe-off phase^26^. To assess functional recovery, the ankle angles during the toe-off phase were evaluated at postoperative weeks 4, 8, 12, and 16. A 1-m-long walking track, 10 cm wide, and 10 cm high, was constructed for this experiment. The video was recorded during the test using a digital camera (SX HS, Canon, Japan) from a distance of 1 m. Recordings were repeated several times for each rat. During the toe-off phase, the angle of the ankle joint was measured at maximal plantar flexion in the experimental lateral ankle joint. After manually identifying the foot and lower leg segments in the video frames, the ankle angles at the toe-off were measured in degrees.

### Isometric tetanic force measurement

The maximum isometric tetanic force was measured at post-operative 16 weeks using previously reported methods^61^. An incision was made in the skin in front of the ankle to expose the distal end of the tibialis anterior (TA) tendon, which was then cut. Stimuli were generated using a bipolar stimulator (Grass Instrument Corp., MA, USA) and processed with LabVIEW software (National Instruments, Austin, TX, USA). The data from the other side were used to normalize the muscle strength, which was then expressed as a percentage of that side’s data.

### Evaluation of tibialis anterior muscle

At postoperative 16 weeks, TA muscles were dissected from the surrounding tissues and weighed on an electronic balance. The weights of TA muscles for each group were photographed and recorded. To compare each group, the weight ratio of the tibialis anterior muscle in the operated area was calculated and compared to the weight ratio of the same muscle in the non-operated area.c

### Contracture ankle angle measurement

The ankle contracture angles were evaluated at postoperative 16 weeks using a previously reported procedure^62^. The angle formed by the anterior border of the tibia and the dorsal aspect of the foot during maximal ankle plantar flexion was measured. The ankle contracture angle was measured by assessing the ankle on the non-implanted side, and the extent of recovery following ankle contracture was compared.

### Analysis of toluidine blue staining

The implanted peripheral nerves were harvested, and fixed using a 2.5% glutaraldehyde solution. The 1-μm thick sections were stained with toluidine blue to visualize myelin under an optical microscope. Images of non-overlapping fields (400 × magnification) were acquired using an upright microscope (BX53, Olympus, Japan) equipped with a CCD camera with 1224 × 960 resolution (cellSens Standard, Olympus, Japan). Image J (National Institutes of Health) was used to analyze the total number of axons in the nerve fibers.

### Analysis of ImmunoHistoChemistry (IHC)

The implanted ANGs were harvested, fixed in 4% PFA, embedded in paraffin, and sectioned into 4-μm slices. The sections were deparaffinized and then rehydrated using a gradient of decreasing ethanol concentrations. The Ultra View peroxidase inhibitor (Roche Diagnostics, USA) was blocked with 3% H_2_O_2_ for 4 min. The sections were incubated with anti-S100β (1:2000 dilution; Abcam, USA) and anti- MBP, 1:5000 dilution; Abcam) at 36 °C for 37 min. A Ventana Benchmark XT device was used for visualizing staining and analysis. Hematoxylin stain was used for the counterstain. The expression of S100β and MBP was visualized using the UltraView Universal DAB Detection Kit (Ventana Medical Systems, Inc. USA). Images of IHC (non-overlapping fields,400 × magnification) were performed using an upright microscope (BX53, Olympus, Japan) equipped with a CCD camera with 1224 × 960 resolution (cellSens Standard, Olympus, Japan). To quantify S100β and MBP, we selected five sections near the transection site from each group and analyzed three fields from each section using Image J software. Representative images are shown in the figures (Supplementry materials).

### Transmission electron microscopy (TEM) analysis

Ultrathin sections of 60 nm thickness were obtained using an ultramicrotome (Ultracut UCT, Leica, Germany). The sections were collected on 200 mesh grids and stained with a 0.2% lead citrate-1% uranyl acetate staining solution. Subsequently, they were examined and photographed using a TEM (HT7800, Hitachi, Japan) operating at 80 kV and a CCD camera. Axon diameters and G-ratios were measured in the electron microscopic images of each group, and the average myelin thickness was calculated. The HITACHI RC16 system was used to measure the myelin thickness in TEM images of each group and to calculate the average thickness.

### Statistical analysis

All experiments were repeated three or more times. All data were presented as mean ± standard error of the mean (SEM). *P*-values less than 0.05 were considered statistically significant. All statistical analyses were performed using the Student’s t-test in GraphPad Prism version 5 (GraphPad Software, Inc., San Diego, CA, USA).

## Supplementary Information

Supplementary Legends

Supplementary Figure 1.

## Data Availability

All data analyzed during this study are available from the corresponding author on reasonable request.
