# Supplementary material for: Increased efficiency of peripheral nerve regeneration using supercritical carbon dioxide-based decellularization in acellular nerve graft
Source: Sci Rep. 2024 Oct 10;14:23696. doi: 10.1038/s41598-024-72672-w (PMC11467423; doi:10.1038/s41598-024-72672-w)
Supplement: Supplementary file 1 — Supplementary Legends [file 41598_2024_72672_MOESM1_ESM.docx]

**Supplementary Fig. 1**. **Characterization of scCO_2_ ANGs.** Decellularization of N (native), Hudson, and scCO_2_ ANGs was verified by detecting the absence of cell nuclei using HE (40´, 100´) and DAPI (100´) staining.
