# Supplementary figures and images for: Increased efficiency of peripheral nerve regeneration using supercritical carbon dioxide-based decellularization in acellular nerve graft
Source: Sci Rep. 2024 Oct 10;14:23696. doi: 10.1038/s41598-024-72672-w (PMC11467423; doi:10.1038/s41598-024-72672-w)

Supplementary Fig. 1.

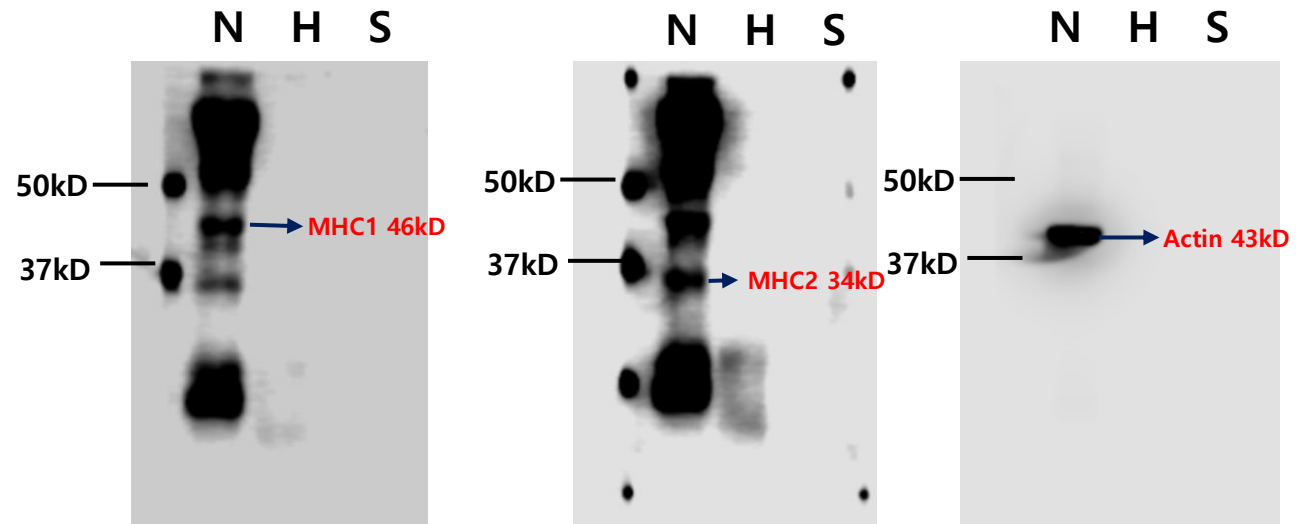

Supplement: Supplementary file 2 — Supplementary Figure 1. [file 41598_2024_72672_MOESM2_ESM.pdf]
